# P-2089. Understanding the impact of socio-structural determinants of health on viral suppression in people living with HIV

**DOI:** 10.1093/ofid/ofaf695.2253

**Published:** 2026-01-11

**Authors:** Alex Olson, Alan Chan, Sergio Romero, Margaret Shea, Clara A Chen, Namkee G Choi, Moka Yoo-Jeong, Archana Asundi

**Affiliations:** Boston Medical Center, Quincy, MA; Boston Medical Center, Quincy, MA; Boston Medical Center, Quincy, MA; Boston University, Boston, Massachusetts; Boston University School of Public Health, Boston, Massachusetts; University of Texas at Austin, Austin, Texas; Northeastern University, Boston, Massachusetts; Boston Medical Center, Quincy, MA

## Abstract

**Background:**

Antiretroviral therapy (ART) has prolonged the lives of people with HIV (PWH), but some individuals continue to face barriers to ART adherence. Socio-structural determinants of health (SsDoH) are often under recognized as important contributors to health and are not often collected. In 2014, the Massachusetts Department of Public Health developed the Acuity Assessment Tool (AAT) for identifying SsDoH to help triage services for PWH. However, its association with viral suppression has not been formally assessed to our knowledge.

**Figure:**
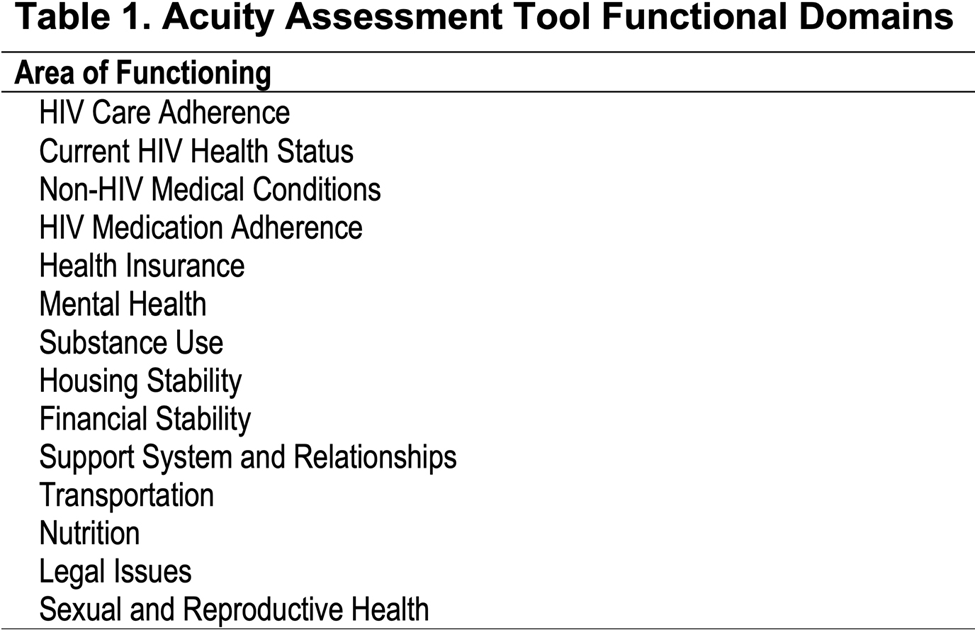

**Figure:**
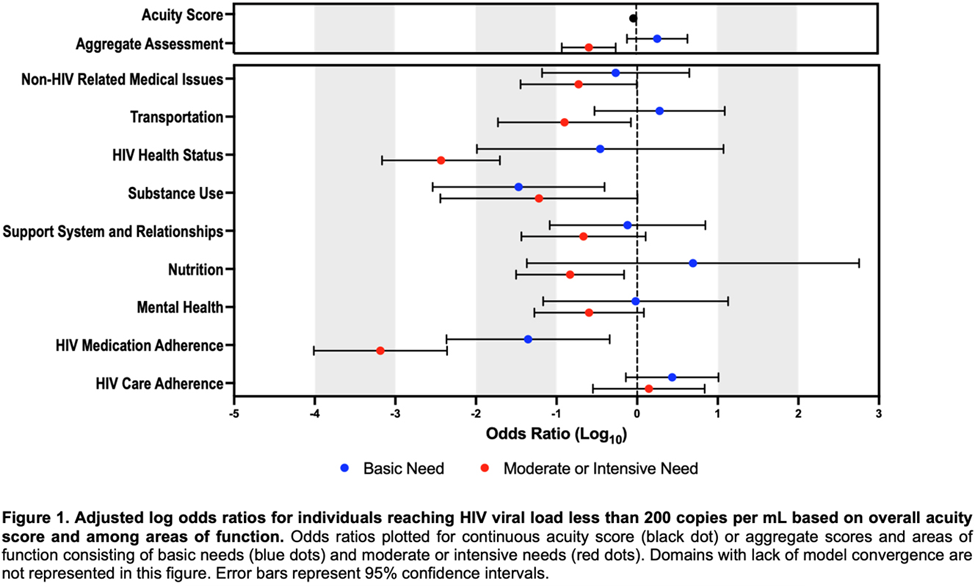

**Methods:**

We conducted a retrospective study using medical record data from PWH receiving care at Boston Medical Center (n=2,236) and included individuals on ART between 3/2022-5/2024 in our analysis (n=1,001). The AAT captures 14 domains of SsDoH (Table 1) and scored on a 4-point scale: self-management (0), basic (1), moderate (2), or intensive (3) need (max score: 42). Viral suppression was defined as < 200 copies/mL. Descriptive statistics along with logistical models were performed to describe the association of AAT scores with viral status.

**Results:**

Among the cohort, the mean age was 53.3 years; 53.4% (n=535) were male, 69.9% (n=700) identified as black, and 68.6% (n=687) were primary English speakers. Approximately 4% (n=41) of individuals had detectable virus. Compared to individuals with viral suppression, those with detectable virus had a higher AAT scores (12.34 vs. 6.65, p = 0.003). In adjusted analyses, those with higher AAT scores and aggregated moderate-to-intensive need were less likely to have viral suppression (Figure 1). Furthermore, domains including HIV care adherence, HIV health status, HIV medication adherence, transportation, substance use, nutrition, and non-HIV medical conditions were associated with lower odds of viral suppression (Figure 1).

**Conclusion:**

Overall, we showed that higher AAT scores associated with poorer viral suppression. Beyond expected HIV-related needs, we identified associations of viral suppression with other socio-structural needs that highlight multifactorial barriers to care. These findings suggest gaps in accessibility and behavioral health management that warrant targeted interventions. Future work is needed to address these gaps and support sustained viral suppression for all PWH.

**Disclosures:**

Archana Asundi, MD, DayZero Diagnostics: Grant/Research Support|Gilead Sciences: Advisor/Consultant|Gilead Sciences: Grant/Research Support|Theratechnologies: Grant/Research Support|Viiv Healthcare: Grant/Research Support

